# Quality preservation and decay reduction of minimally processed seedless barberry fruit via postharvest ultrasonic treatment

**DOI:** 10.1002/fsn3.3698

**Published:** 2023-09-18

**Authors:** Mohammad Reza Saebi, Farid Moradinezhad, Elham Ansarifar

**Affiliations:** ^1^ Department of Horticultural Science, Faculty of Agriculture University of Birjand Birjand Iran; ^2^ Department of Public Health, School of Health, Social Determinants of Health Research Center Birjand University of Medical Science Birjand Iran

**Keywords:** antioxidant, decay, postharvest, seedless barberry, ultrasound

## Abstract

Seedless barberry fruit is one of the important horticultural products of Iran, which has health benefits due to great amounts of phenolic compounds, flavonoids, and antioxidant activity. However, fresh barberry fruit has a short shelf life even at cold storage, mainly due to high water content and thin skin that leads to fungal decay and high postharvest loss. We examined the effectiveness of the postharvest ultrasonic technology on the quality preservation and nutritional value of fresh seedless barberry fruit and their decay reduction during cold storage. Experimental treatments were the time and temperature of ultrasound (US) and included: (1) control, fruit without US, (2) 5 min US at 20°C, (3) 5 min US at 30°C, (4) 5 min US at 40°C, (5) 10 min US at 20°C, (6) 10 min US at 30°C, (7) 10 min US at 40°C, (8) 15 min US at 20°C, (9) 15 min US at 30°C, and (10) 15 min US at 40°C. After applying the treatments, the fruits were sealed in polyethylene bags and stored at 4 ± 1°C for 20 days. The results showed that all US treatments had higher titratable acidity, antioxidant activity, phenol content, and vitamin C content than the control. However, the highest titratable acidity and antioxidant activity values were obtained in US treatments at 40°C and 30°C for 15 min. Also, US treatment significantly reduced the total soluble solids, decay percentage, and microbial load of fresh barberry fruit. As the US treatment temperature increased from 20°C to 40°C, the decay and microbial load of fruit significantly decreased. In conclusion, this study proved the potential application of the US for preserving the quality of fresh seedless barberry fruit, and the most optimal US temperature and its application time was 40°C for 15 min.

## Graphical abstract

All US treatments had higher titratable acidity, antioxidant activity, phenol content, and vitamin C content than the control. US treatment significantly reduced the total soluble solids, decay percentage, and microbial load of fresh barberry fruit. In conclusion, this study proved the potential application of US for preserving the quality of fresh seedless barberry fruit.

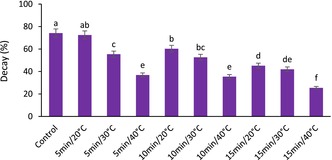

## 1 INTRODUCTION

Processing methods affect the nutritional quality, safety, and shelf life of food (Moradinezhad et al., 2020). The thermal process guarantees the food's health and improves its shelf life, but it hurts its nutritional properties (Gómez et al., 2011). For this reason, researchers are seeking new processing methods without applying heat or with gentle heat (Moradinezhad et al., 2018; Ranjbari et al., 2018). Ultrasound (US) technology provides a non‐destructive, fast, and reliable technique for relating the indicators and characteristics of the specific quality of fruits and vegetables during growth, maturity, and storage until they are ready to be consumed (Mizrach, 2008). The US consists of sound waves with a frequency beyond the range of human hearing (generally beyond 20 kHz). The techniques of using the US are relatively cheap, simple, and with low energy consumption, and today, this advanced technology has become typical for research and improvement of food and agricultural products (Bhargava et al., 2021; Rahimi et al., 2022).

The effect of optimizing ultrasonic waves to increase shelf life and maintain quality characteristics postharvest has been reported in many agricultural products, so that in most cases, it has superior effects compared to other common methods (Ganjdoost et al., 2021; Hashemi et al., 2020; Xu et al., 2022). For example, in a study on strawberries, the effect of optimizing the US waves on fruit decay and the physiological quality of fruit was investigated (Cao, Hu, & Pang, 2010). The results showed that the ultrasonic treatment of 40 kHz at a temperature of 28°C and a duration of 10 min significantly (*p* < .05) reduces decay and the number of microorganisms. The US also prevented the reduction of firmness and maintained a significant level of total soluble solids, titratable acidity, and vitamin C in peach fruit (Cao, Hu, Zheng, & Lu, 2010).

Barberry is a thorny shrub native to the northern Himalayas and is widely distributed in tropical Asia, South America, Africa, and some parts of Europe (Sarraf et al., 2019). Most barberries (*Berberis vulgaris* L.) cultivated in Iran are seedless and edible, although there are some seedy varieties as ornamental and medicinal plants. The berries of seedless barberries are consumed as a dessert and contain significant amounts of antioxidants and minerals (Moradinezhad, Mehregan, & Jahani, 2019). Major minerals in the fruit include zinc, iron, magnesium, and potassium. The fruit is also rich in phenolic compounds and flavonoids (Hosseini et al., 2022). Alkaloids in barberry, along with phenolics and flavonoids, have anti‐diabetic, liver‐protective, immune system‐modulating, anti‐cancer, anti‐microbial, anti‐inflammatory, antioxidant, and anti‐diarrheal properties (Kumar et al., 2022). The fresh fruit (berry) of barberry contains much water and thin skin in the ripening stage. Therefore, it is susceptible to bursting and spoilage during different stages of harvesting, handling, storage, and processing (Aleissa et al., 2023). Thus, different postharvest treatments are considered to increase shelf life and maintain the nutritional value of various fresh fruit (Moradinezhad et al., 2013; Moradinezhad, Ghesmati, & Khayyat, 2019).

Although the beneficial effects of postharvest application of the US in food and agriculture industries indicated, no data are available regarding the effects of the US on the postharvest quality attributes and shelf life of fresh seedless barberry fruit at cold storage. Therefore, the purpose of this research was to investigate the potential of US treatment (different times and temperatures) in extending the shelf life and maintaining the quality characteristics of fresh seedless barberry fruit during cold storage.

## 2 MATERIALS AND METHODS

### 2.1 Preparation of berries and US treatment

The seedless barberry fruits were harvested after fully ripening in November 2020 from a private garden in the suburbs of Qaen (33° 73′ N, 59° 17′ E), South Khorasan Province, Iran. Immediately after separating the berries from branches, they were transferred to the laboratory of Birjand University of Medical Sciences for US treatment. Treated samples were then packed and transferred to the Horticulture Laboratory of Birjand University, where they were stored at 4°C until the end of the storage period for physicochemical measurements.

The fresh barberry fruit samples were treated with ultrasonic waves (SONOREX SUPER RK 510H model, BANDELIN electronic GmbH & Co. KG) at 35 kHz power, and at three different temperatures (20, 30, or 40°C) for varying treatment times (5, 10, or 15 min). Treatments included: (1) Control, fruit without US, (2) 5 min US at 20°C, (3) 5 min US at 30°C, (4) 5 min US at 40°C, (5) 10 min US at 20°C, (6) 10 min US at 30°C, (7) 10 min US at 40°C, (8) 15 min US at 20°C, (9) 15 min US at 30°C, and (10) 15 min US at 40°C.

After applying the treatments, 50 g of fruit were sealed in a transparent polyethylene bag as a replicate and stored for 20 days at 4°C. The quality traits of the berries were evaluated at the end of the storage period.

### 2.2 Measurement of quality characteristics

Total soluble solids (TSS) were read by placing 1 to 2 drops of filtered barberry water on the prism plate of a manual refractometer model (RF10, 0–32 °Brix, Extech, USA) and expressed in °Brix. To measure the titratable acidity (TA), 4 mL of fruit juice (with a dilution of 20) was diluted. Finally, the titration was done with 0.1 Normal NaOH solution to pH = 8 (Sadler & Murphy, 2010). The antioxidant activity of the fruit extract was determined through the free radical scavenging method (DPPH). Free radical scavenging activity (total antioxidant) was measured using the 2‐2‐diphenyl‐2‐picrylhydrazyl (DPPH) method and expressed as a percentage (Yu et al., 2021). The results are expressed as DPPH radical inhibition percentage using Equation (1):

(1) $$\text{DPPH radical scavenging activity}\%=\left(1-{\mathrm{Abs}}_{\text{sample}}/{\mathrm{Abs}}_{\text{control}}\right)\times 100$$

where Abs_sample_ and Abs_control_ represent the absorbance of the sample and control, respectively.

Total phenol content was calculated using the gallic acid method and Folin–Ciocalteu reagent through spectrophotometry (Nikolaeva et al., 2022). The wavelength used to measure the phenol content was 725 nm, and the results were finally calculated in terms of milligrams of gallic acid per 100 grams of fresh weight. The evaluation was based on the *standard curve* of *gallic acid*.

The amount of ascorbic acid was measured by the titration method with sodium 2, 6‐dichlorophenol‐indophenol and expressed in terms of mg of ascorbic acid per 100 grams of sample (Hughes, 1983). Ion leakage in fruit was measured using the primary and secondary electrical conductivity (EC) method (Saltveit, 2002). After the storage period, the fruit decay was examined visually in terms of the presence of fungal, bacterial surface contamination, chilling injury, or discoloration of the fruit surface, and it was calculated as a percentage (Mamatha et al., 2000). Since the berries are small in size, when decay symptoms were observed on the fruit (regardless of the level of decay), that fruit was considered decayed (Dorostkar et al., 2022). The colony counting method was used to investigate microbial populations on barberries (Ansarifar & Moradinezhad, 2022). Total bacteria and total fungi and yeast were estimated on the plate count agar (PCA) incubated at 37°C for 2 days, and on potato dextrose agar (PDA) incubated at 28°C for 3 days, respectively.

### 2.3 Statistical analysis

The statistical design used in this research was a completely randomized design. Three replications (10 treatments × 3 replications) were considered for each treatment. Statistical data analysis was done by SAS version 9.3, and the mean comparison between treatments was calculated with LSD test at 5% probability level.

## 3 RESULTS AND DISCUSSION

### 3.1 Total soluble solids

**FIGURE 1:**
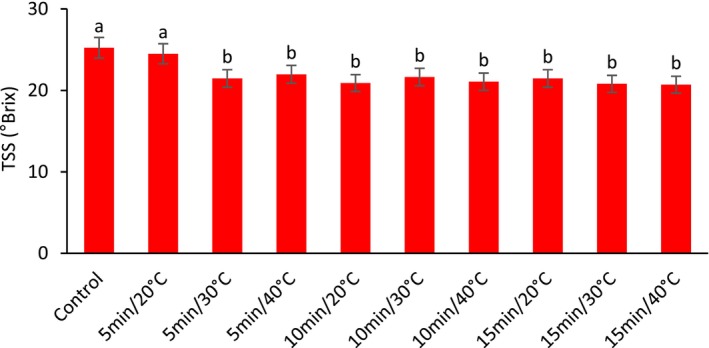
The effect of different ultrasonic temperatures and times on the total soluble solids (TSS) of fresh seedless barberry stored for 20 days at 4°C. Means followed by similar letters are not significantly different according to least significant difference (LSD) test (*p* ≤ 0.05).

The results showed that US treatment at different times and temperatures affected the total soluble solids values in fresh barberry. According to Figure 1, the highest level of TSS was obtained from control and ultrasonic treatment at 20°C for 5 min, and the lowest amount was observed in the US treatment at 40°C for 15 min. However, there was no significant difference between the US treatment at 40°C for 15 min. and other treatments (except the control treatment and 5 min at 20°C).

TSS are temporary energy storage mainly involved in carbohydrate metabolism in cells and are considered an indicator of post‐harvest spoilage (Jiang, 2013). As shown in Figure 1, TSS significantly decreased in all US treatments compared to the control except at 20°C for 5 min treatment. The effectiveness of US treatment strongly depends on the frequency, power of the sound waves applied, time, and temperature of the treatment.

In the process of ripening, polymeric carbohydrates, especially sugars in the cell wall, are broken and converted into simpler compounds, which cause changes in the taste and texture of the product. For this reason, the amount of soluble solids increases with the ripening of the fruit (Wurochekke et al., 2013). It is well known that the synthesis of polygalacturonase (PG) and pectin methylesterase (PME) enzymes are among the key enzymes in the degradation of carbohydrates (Li, Bai, et al., 2022). Ultrasonic treatment likely inhibits the activity of these enzymes (Cao, Hu, Pang, Wang, et al., 2010). Similar to the results of the current research, Lopez et al. (1998) showed that US can cause structural changes in enzymes and especially the inactivation of PME enzyme in tomato juice. The mechanism of US action on enzyme deactivation can be described as mechanical; cavitation caused by US causes changes (such as pressure, temperature, stress, and pH) in the environment around the enzyme (Li, Deng, et al., 2022). The shear force produced by the ultrasonic cavitation bubbles can break the hydrogen bond, vanderwaals, hydrophobic interaction, and electrostatic force that maintain the stability of the spatial structure of the protein and thus change the secondary and tertiary structures of the enzyme (Larsen et al., 2021). These changes consequently lead to denaturation and inactivation of the enzyme (Iqbal et al., 2019; Sánchez‐Rubio et al., 2018). Therefore, in the current research, the reduction of TSS can probably be related to the property of enzyme inhibition by US treatment. On the other hand, free radicals produced by water sonolysis (H_2_O → OH^•^ + H^•^) interact with amino acid residues and affect enzyme activity (Jiang et al., 2020).

### 3.2 Titratable acidity

**FIGURE 2:**
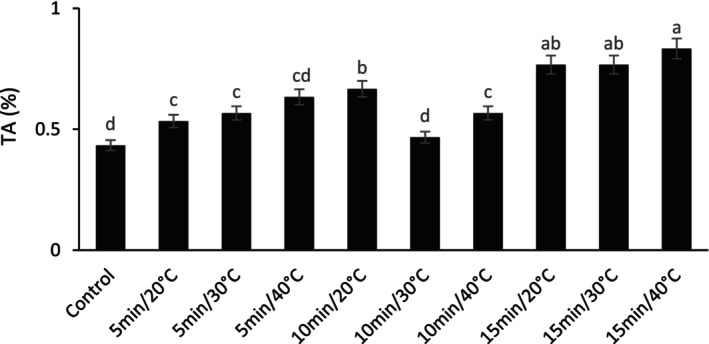
The effect of different ultrasonic temperatures and times on the titratable acidity (TA) of fresh seedless barberry stored for 20 days at 4°C. Means followed by similar letters are not significantly different according to least significant difference (LSD) test (*p* ≤ 0.05).

The titratable acidity of fresh barberry fruit was affected by US treatments. After 20 days of cold storage, the highest and lowest TA were obtained from US treatment at 40°C for 15 min and control, respectively (Figure 2). However, there was no significant difference between the 15 min at 40°C and 15 min at 20°C or 30°C treatments. However, TA was about 40% higher in the 15 min at 40°C treatment than in the control.

Titratable acidity is directly related to the concentration of dominant organic acids in the fruit, an essential parameter in fruit quality (Masithoh et al., 2016). Since organic acid is used as a substrate for the enzymatic reactions of respiration, the fruit's acidity should decrease during the postharvest period (Etienne et al., 2013). Therefore, likely any treatment that can reduce the respiration rate and ethylene production causes the accumulation of titratable acidity in the fruit tissue. Mustapha and Zhou (2021) showed that ethylene production decreases with the application of US on cherry tomatoes. They stated that the decrease in ethylene production may be due to the US process, which stops the enzyme activities responsible for ethylene synthesis. The results of the present research showed that titratable acidity is optimally maintained by increasing the temperature and US time, which is likely due to the decrease in ethylene production and inhibition of respiration as a result of US treatment. Our results were in line with the findings of other researchers on guava (Kalsi et al., 2022), strawberry (Maryam et al., 2021), and pomegranate fruit (Moradinezhad et al., 2023).

### 3.3 Antioxidants activity

**FIGURE 3:**
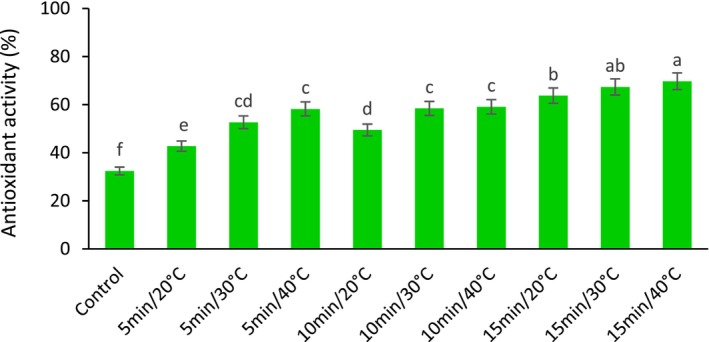
The effect of different ultrasonic temperatures and times on the antioxidant activity of fresh seedless barberry stored for 20 days at 4°C. Means followed by similar letters are not significantly different according to least significant difference (LSD) test (*p* ≤ 0.05).

The results showed that the antioxidant activity significantly increased with increasing temperature and time of US in all treated samples than control. The highest amount of fresh barberry antioxidant was obtained from US treatment at 40°C for 15 min (15 min at 40°C), and the lowest was related to control fruit. However, no significant difference was found between the 15 min at 40°C and 30°C treatments (Figure 3).

Recent findings have indicated that fruit senescence is related mainly to reactive oxygen species (ROS) and oxidative damage to proteins in mitochondria (Tian et al., 2013). The enzyme system that suppresses oxygen free radicals, including the enzymes superoxide dismutase (SOD), catalase (CAT), peroxidase (POD), and other enzymes, plays a vital role in protecting plants against oxidative stress (Jajic et al., 2015). Wang et al. (2015) investigated the activity of these three enzymes (CAT, POD, SOD) in US‐treated cherry tomatoes during storage. They showed that the CAT, POD, and SOD activities of cherry tomatoes were significantly higher than the control after 16 days of storage, which indicated the higher antioxidant activity of the US‐treated fruits. In in vitro enzyme reaction systems, power of the US can be used to have a positive effect on enzyme activity, as it improves the efficiency of enzyme reactions (Fan et al., 2021). However, when it comes to multicellular tissues of fruits and vegetables, the response to the US may be much more complex. Few studies have reported the effects of the US on the enzymatic activity of fruits and vegetables after harvest (Rajaei et al., 2021). Wei and Ye (2011) found that US treatment enhanced the effect of 6‐benzylaminopurine (6‐BAP) on asparagus during storage, thereby increasing the CAT activity of asparagus, which caused increases in antioxidant activity. As mentioned, the cavitation process resulting from US causes the production of free radicals from the water sonolysis reaction. It has also been suggested that production of hydroxyl radicals by hydroxylation of food components by ultrasonic waves can also increase its antioxidant activity (Ashokkumar et al., 2008).

### 3.4 Total phenol content

**FIGURE 4:**
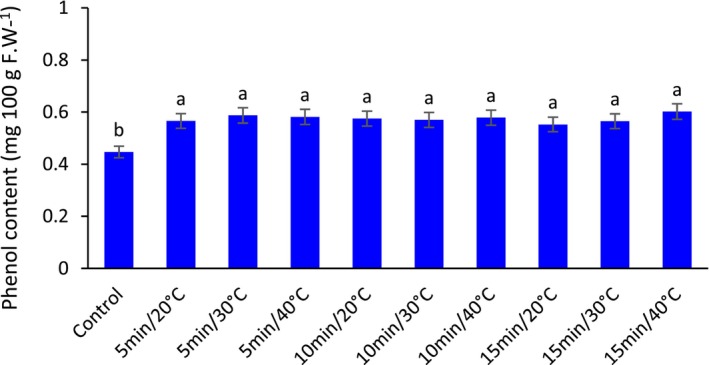
The effect of different ultrasonic temperatures and times on the total phenol content of fresh seedless barberry stored for 20 days at 4°C. Means followed by similar letters are not significantly different according to least significant difference (LSD) test (*p* ≤ 0.05).

According to Figure 4, US significantly increased total phenol content in fresh barberry fruit of all US treatments compared to the control. However, there was no significant difference between US treatments.

Phenolic compounds are essential and valuable for human health due to their significant role in reducing the risk of physiological diseases in humans (Ho et al., 2020). The phenolic compounds in healthy fruit are oxidized to ortho‐quinones in case of cell damage and exposure to oxygen, and their amount decreases significantly (Takahama & Hirota, 2017). This oxidation is done by some enzymes, such as polyphenol oxidase (PPO) enzyme (Michalík et al., 2018). The effect of this oxidation appears in the form of brown and dark colors. Chen et al. (2012) showed that US (40 kHz), with a power of 120 W on litchi fruit, decreased the activity of the polyphenol oxidase enzyme by reducing the active substrate of this enzyme. There was significantly more total phenolic content in US‐treated fruit than in untreated samples, which is consistent with the results of the present study.

Another enzyme that is effective in the phenolic content of fruit is phenylalanine ammonia‐lyase (PAL). Yeoh and Ali (2017) showed a strong correlation (*r* = .7528, *p* < .001) between PAL enzyme and phenol content in sliced pineapple fruit. Yang et al. (2011) also showed that the combination of US treatment and salicylic acid could provide a higher defense capacity against post‐harvest rot caused by *Penicillium expansum*, as this treatment increases the PAL enzyme and thus treated fruit has a higher phenolic content, and also a higher defensive capacity. Similarly, the US has been recommended to increase the accumulation of phenolic compounds in pear juice (Zafra‐Rojas et al., 2013). In another study on sliced pineapple fruit (Yeoh & Ali, 2017), it was shown that US treatment increases the content of total phenol in fruits. However, they reported that increasing the US output power significantly decreased the total phenolic content of sliced pineapple after 5 days of cold storage. It should be noted that US waves with large amplitudes can facilitate the displacement of molecules and collapse pressure, enhancing the formation of free radicals in the aqueous solution that causes cell destruction (Sauter et al., 2008).

### 3.5 Vitamin C

**FIGURE 5:**
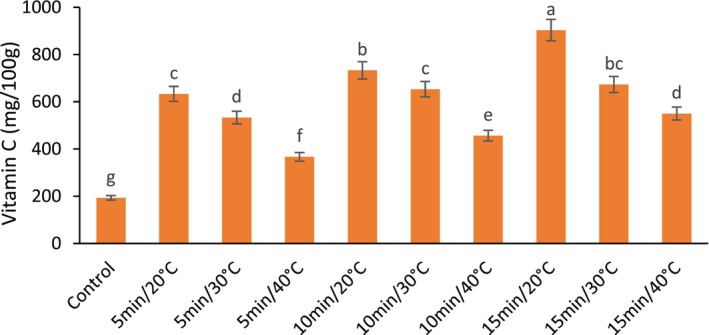
The effect of different ultrasonic temperatures and times on the Vitamin C (Ascorbic acid) of fresh seedless barberry stored for 20 days at 4°C. Means followed by similar letters are not significantly different according to least significant difference (LSD) test (*p* ≤ 0.05).

Postharvest application of the US had a significant effect on the vitamin C content of fresh barberry fruit. US treatment significantly enhanced vitamin C content in all treatments compared to the control. The highest and lowest amount of vitamin C was recorded from US treatment at 20°C for 15 min and the control, respectively. Regardless of US time, the vitamin C content decreases with the increase in temperature in the US treatment (Figure 5). In addition, with increasing the US time from 5 to 15 min, the vitamin C values in barberry fruit significantly increased.

Vitamin C content in fruits and vegetables is often used to indicate the overall nutritional quality of food products (Fenech et al., 2019). During evolution, some animals, as well as humans, have lost the ability to synthesize ascorbic acid (ascorbate, vitamin C), an essential molecule in the physiology of animals and plants (Caritá et al., 2020). In addition to its primary role as an antioxidant and cofactor in redox reactions, recent reports have shown the essential role of ascorbate in the activation of epigenetic mechanisms controlling cell differentiation, whose dysregulation can lead to the development of certain types of cancer (Caritá et al., 2020).

L‐ascorbic acid (L‐threo‐hex‐2‐enono‐1,4‐lactone, ascorbate), also called vitamin C, plays many roles in plant cells. The important properties of vitamin C are its antioxidant capacity and the completion of oxidative chain reactions that lead to the production of non‐oxidative products such as dehydroascorbate (DHA) and 2,3‐dictogulonic acid (Davey et al., 2000).

It has been proven that hydrogen peroxide (H_2_O_2_) plays an essential role in plant growth and defense (Brudzynski, 2020; Nazir et al., 2020), and it can be found in different organs of plant cells (Exposito‐Rodriguez et al., 2013). However, H_2_O_2_ is also partially responsible for light‐induced oxidative damage. Ascorbate (vitamin C) is involved in removing excess H_2_O_2_ produced during photosynthesis under high irradiance conditions by the action of ascorbate peroxidases (APX), enzymes that are absent in animals (Wheeler et al., 2015). The results of the current research showed that US treatment preserves vitamin C. As mentioned earlier, the US enhances the enzymatic antioxidant system. Likely, with the increase of antioxidant enzymes by the US, less ascorbate is used to neutralize free radicals. For this reason, the use of US leads to the accumulation of vitamin C in plant tissue. However, it should be noted that losses of this vitamin are mainly attributed to its solubility in water and its sensitivity to high temperatures and oxidation conditions (oxygen, pH, and metal ions) (Davey et al., 2000).

### 3.6 Ion leakage

Evaluation of ionic leakage in fresh barberry fruit showed that it was not affected by US treatments during the storage period. The ion leakage percentage in the present experiment ranged between 30% and 35%.

Measurement of ion leakage is a helpful tool for predicting changes in intracellular structure and the extent of cell damage in plant tissues (Sevik & Karaca, 2016). When a cell dies and loses its membrane integrity, electrolytes such as K^+^ ions leak out. Therefore, we can use the number of electrolytes leaked from the tissue as a proxy for the amount of cell death in the tissue (Yılmaz & Bilek, 2018).

It has been shown that the application of US increases cell permeability and cell wall rupture in plant tissues (Mekhilef et al., 2012). Cavitation can be the leading cause of increased cell rupture. The sponging effect (or cavitation) can also lead to the formation of microscopic channels in fruit tissues (Yao, 2016), thereby leading to increased ion leakage values in sonicated samples. Yılmaz and Bilek (2018) showed that increasing the power of US (198w) increased the ion leakage of apple discs by 45%. However, in the current research, it was found that the application of US on fresh barberry fruit with a frequency of 35 kHz (for 5, 10, and 15 min) and at different temperatures (20, 30, and 40°C) did not hurt the cell wall, and so did not cause ion leakage. In line with the results of our study, Wiktor et al. (2016) did not observe any significant difference in the electrical conductivity of apple discs that were processed at frequencies of 21 or 40 kHz and 180 watts for 5, 10, 20, and 30 min. They concluded that, unlike the treatment of pulsed electric fields, the application of US does not damage plant tissues.

### 3.7 Decay percentage and microbial analysis

**FIGURE 6:**
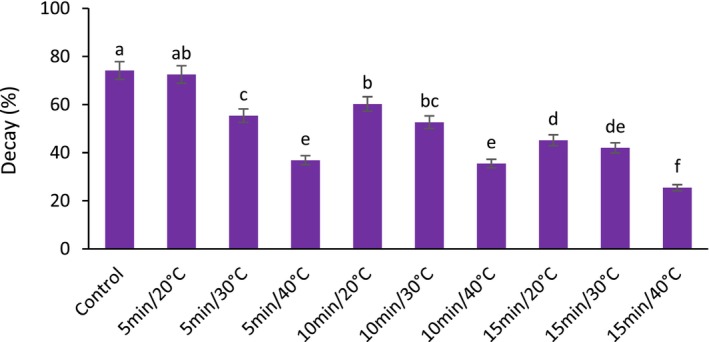
The effect of different ultrasonic temperatures and times on the decay of fresh seedless barberry stored for 20 days at 4°C. Means followed by similar letters are not significantly different according to least significant difference (LSD) test (*p* ≤ 0.05).

US treatments significantly decreased fruit decay compared to the control except at 20°C for 5 min treatment. The results showed that the fresh fruit decay varied from 20 to 80% during the cold storage period. The highest percentage of decay was related to control fruit. Also, the lowest decay percentage was observed in 15 min at 40°C US treatment. According to Figure 6, with the temperature increase in the US process, the decay percentage significantly decreased.

The results of the microbial analysis also showed that as US temperature and its application time increased, the growth rate of bacteria, fungi and yeast on fresh barberries samples was significantly affected and reduced in all US treatments compared to the control. The lowest bacterial count (1.12 log CFU/g) was observed in US treatment at 40°C for 15 min, whereas the highest (6.85 log CFU/g) was found in the control sample. Similarly, the lowest yeast and mold count (2.53 log CFU/g) was obtained in US treatment at 40°C for 15 min, and the highest (7.62 log CFU/g) was observed in control fruit.

Microbial infection is the main cause of postharvest rotting of fruits and vegetables. In addition, microorganisms such as *Salmonella*, *E. coli*, etc., on the surface of fresh vegetables and fruits, make possible the spread of foodborne diseases (Goodburn & Wallace, 2013; Neto et al., 2019). Therefore, it is necessary to develop an effective method to control microbial activity and reduce the spread of foodborne diseases. In this regard, ultrasonic technology is an effective auxiliary sterilization method (Chen et al., 2020; Wang et al., 2020). It is suggested that the inactivation of the pathogen by the US is primarily due to the phenomenon of cavitation (sponging), because in this phenomenon, bubbles are formed, then, due to the collapse of these bubbles, the temperature and pressure increase. Consequently, this process destroys the microorganisms (Chen et al., 2020). The resulting massive hydraulic shock wave may damage pathogen cell walls and cytoplasmic membranes (Sienkiewicz et al., 2017). Another mechanism of the US is that free radicals produced by the explosion of bubbles enter the cell, react with the internal components, and destroy the cell (Liao et al., 2018). As a non‐thermal sterilization technology, ultrasonic sterilization treatment has attracted the attention of researchers in the field of postharvest storage of fruits and vegetables. In the current research, it was found that by applying US at a temperature of 40°C and its application time of 15 min, the decay percentage and overall microbial count decreased significantly compared to the control, which is in accordance with the literature presented.

Studies have been conducted on ultrasonic treatment of freshly harvested strawberries to reduce the microbial population (Cao, Hu, & Pang, 2010). While reducing strawberry microbial load, ultrasonic treatment can maintain fruit firmness and improve fruit antioxidant activity. In a study by Pinheiro et al. (2015), the effect of US treatment on postharvest quality and microbial load of tomato fruit during storage was evaluated. The results showed that the US can significantly reduce the primary mesophyll load immediately after sonication under two processing parameters (80% power level—15 min, 100% power level—19 min). It is worth noting that the firmness of US‐treated tomatoes remained similar to untreated samples. In addition to the ultrasonic power level and processing time, the effect of ultrasonic processing is also affected by storage temperature and other environmental conditions. It was observed that the antibacterial effect of US on pathogenic bacteria was significantly affected by the storage temperature (Neto et al., 2019).

## 4 CONCLUSION

In general, the results of this study indicated that the US has the potential to use it as a post‐harvest eco‐friendly treatment for quality preservation of fresh seedless barberry fruits. US treatment at a proper time and temperature had positively impacted on the fruit quality. The total soluble solids and total phenol content were preserved in the US‐treated fruit compared to the control. In addition, by increasing US treatment temperature and its application time, titratable acidity and antioxidant activity significantly increased and the fruit decay and microbial load significantly reduced. However, increasing the temperature in the US treatment (40°C) reduced the vitamin C content. Although, increasing the US time from 5 to 15 min increased the amount of vitamin C. US treatments also did not increase ion leakage. Therefore, US treatments in the present research do not cause any damage to the seedless barberry fruit tissue. In general, it can be concluded that the optimization of ultrasonic treatment for fresh seedless barberry fruit at temperature of 30 or 40 degrees Celsius for 15 min had the best results from post‐harvest viewpoint.

## AUTHOR CONTRIBUTIONS

**Mohammad Reza Saebi:** Formal analysis (equal); investigation (equal); methodology (equal); writing – original draft (equal). **Farid Moradinezhad:** Methodology (equal); writing original draft (equal); writing – review & editing (equal). **Elham Ansarifar:** Investigation (equal); methodology (equal); writing – review & editing (equal).

## FUNDING INFORMATION

Authors would like to thank gratefully the University of Birjand for providing the financial support of this project.

## CONFLICT OF INTEREST STATEMENT

The authors declare that they have no conflict of interest.

### ETHICS STATEMENT

The corresponding author will follow the ethical responsibilities of authors and COPE rules. This study does not involve any human or animal testing.

### CONSENT TO PARTICIPATE

On behalf of all co‐authors I (Farid Moradinezhad) believe the participants are giving informed consent to participate in this study.

### CONSENT FOR PUBLICATION

I, Farid Moradinezhad, give my consent for submitted manuscript to be published in the Food Science & Nutrition.

## Data Availability

All data are presented in the manuscript.
